# Prognosis of COVID-19 in respiratory allergy: a systematic review and meta-analysis

**DOI:** 10.1186/s43168-022-00110-4

**Published:** 2022-03-04

**Authors:** Alia Abdelmonem El Shahawy, Kelechi Elizabeth Oladimeji, Aboalmagd Hamdallah, Amal Saidani, Rami Abd-Rabu, Nesrine Ben Hadj Dahman

**Affiliations:** 1 Department of Microbiology and Immunology, Faculty of Medicine, Zagazig University, Zagazig, Egypt; 2 Department of Public Health, Faculty of Health Sciences, Walter Sisulu University, Eastern Cape, South Africa; 3 College of Graduate Studies, University of South Africa, Pretoria, South Africa; 4 Faculty of Medicine, Al-Azhar University, Damietta, Egypt; 5 Pulmonology Department, Abderrahmen Mami Hospital, Aryanah, Tunisia; 6 Faculty of Medicine, University of Tunis El Manar, Tunis, Tunisia; 7 Robert D. and Patricia E. Kern Center for the Science of Healthcare Delivery, Mayo Clinic, Rochester, MN USA

**Keywords:** COVID-19, Prognostic outcomes, Respiratory allergy

## Abstract

**Background:**

Do underlying allergic respiratory diseases such as asthma and rhinitis predispose to a severe coronavirus (COVID-19) infection? We conducted this systematic review to map out and synthesize evidence of published literature.

**Main body of the abstract:**

We searched five bibliographic databases for articles published between 1 January and 15 November 2020 using keywords: “COVID” AND “Allergic disease,” “Prognosis and COVID-19,” “SARS-CoV-2,” “Asthma,” “Allergic rhinitis.” We synthesized 32 eligible articles from a total of 11,376 articles retrieved from the search process. The profile of allergic respiratory conditions was identified, and only seven studies reported on the treatment administered. No significant difference was observed concerning the prevalence of COVID-19 in individuals with allergic asthma and those with non-allergic asthma (RR = 0.61, *p* = 0.08). The mortality rate significantly decreased in COVID-19-infected patients with asthma than patients without asthma (RR = 0.63, *p* = 0.04).

**Short conclusion:**

There is little evidence available on the role of asthma medications and risk factors influencing the prognostic outcomes for COVID-19 individuals with respiratory allergies, which invites further research.

## Background

Respiratory allergy, which infers that IgE-mediated allergic reaction is the major underlying pathophysiology in the upper and lower airways, includes allergic rhinitis and asthma [1–3]. In allergic individuals, airways exposure to an allergen will provoke allergic rhinitis and asthmatic reaction [4]. As is typical of most respiratory viruses, the main entry point in the human body by the on-going novel coronavirus disease (COVID-19), announced a global pandemic, is through the nose and nasopharynx airway passage [5]. The main clinical features of COVID-19 are such that respiratory allergic diseases like allergic rhinitis and asthma mimic symptoms of COVID-19, runny nose and headache, are common symptoms of allergic rhinitis, while cough and dyspnea are shared with asthma [6]. Furthermore, one of the prevailing comorbidity conditions identified in individuals infected with COVID-19 is chronic respiratory diseases like respiratory allergies [3].

The chronic allergic disease is linked to the tissue remodeling process, and persistent inflammation with characteristic CD4 T helper 2 (Th2) polarization can impair the efficient antiviral immune response [7]. In that regard, Th2 cytokines have been implicated in the viral progression due to their suppressive effect on physical, humoral barriers against viruses [8]. Further findings have shown the role of Th2 cytokines in coronavirus recognition and infection through modulation of the angiotensin-converting enzyme-2 (ACE2) in the airways and transmembrane protease, serine2 (TMPRSS2) [9, 10].

However, it is still unclear whether respiratory allergies such as asthma and rhinitis predispose one to rapid infection with COVID-19, or whether COVID-19 raises the risk of distressing respiratory allergies [11]. In addition to this research gap on the causal relationship between COVID-19 and respiratory allergies, there is currently no internationally approved therapies or vaccine for clinical trials that can be used to effectively manage COVID-19 infection particularly in adults and children with respiratory allergies [12]. Although informed severity of COVID-19 in children is minimal compared to adults, there is no evidence that allergic rhinitis and asthma medicines currently available, including inhaled corticosteroids (ICSs), antihistamines, and bronchodilators, increase the severity of COVID-19 infection in both adults and children with respiratory allergies [11].

Likewise, there is limited published evidence on the prognostic outcomes for COVID-19 in individuals with respiratory allergies [12]. One of the few available evidences is that from a nationwide cohort study from South Korea, which demonstrated that both allergic rhinitis and asthma were associated with worse clinical findings in individuals infected with COVID-19. Remarkably, patients with non-allergic asthma had a greater risk of testing positive for SARS-CoV-2 test and having a severe prognosis than patients with allergic asthma [13]. Given these uncertainties and limited evidence on clinical outcomes for COVID-19 in individuals with respiratory allergies, we conducted this systematic review to map out evidences and report findings from the synthesis of published literatures. Our review question and objective are outlined below.

## Main text

### Review questions

1. Does underlying respiratory allergic disease increase risk of COVID-19 infection?
2. What are the prognostic outcomes of COVID-19 infection in individuals with respiratory allergic diseases? Does Allergy medication affect the prognostics?

### Main objective

To determine risk factors for COVID-19 and the prognostic outcome in patients with respiratory allergic conditions.

## Methods

### Study design

This systematic review and meta-analysis were conducted and reported according to the Preferred Reporting Items for Systematic Reviews and Meta-Analyses [14] and Meta-analysis Of Observational Studies in Epidemiology (MOOSE) [15] guidelines. The registration number for this review protocol in the International Prospective Register of Systematic Reviews (PROSPERO) is CRD42020198329.

### Search sources and strategy

We carried out a comprehensive search on PubMed/MEDLINE, Web of Science, Google Scholar, and EBSCO using a search strategy that was developed by two of our reviewers (AAES and BA). The search strategy contained medical subject headings (MeSH) and keywords that include “COVID” AND “Allergic disease,” “Prognosis and COVID-19,” “2019-nCoV,” “coronavirus,” “SARS-CoV-2,” “Asthma,” “Allergy,” “Allergic rhinitis,” and “COPD”. The time frame for the search process was between 1 January 2019 and 15 November 2020.

### Database screening

The database retrieved from the search process was reviewed for the removal of duplicates by AAES. The initial search process was conducted in June 2020 and thereafter, another search was conducted 15 November 2020. Two other authors (AS and AH) independently carried out the title and abstract screening of the articles in the first database while AAES and RA independently screened the newly retrieved database. To ensure that the methodological rigor is maintained during the screening process, NBHD independently reviewed the database after the title and abstract screening has been carried out. Lastly, full text of identified eligible studies from the title and abstract screening process were evaluated to determine articles were finally selected for data synthesis.

### Study selection process

The titles and abstracts were screened and evaluated for selection into the study using our eligibility criteria which included the population (patients with respiratory allergic conditions infected with COVID-19), exposure (respiratory allergic diseases and their medication), comparison (if data available-compare outcome among COVID-19 infected individuals with or without respiratory allergy diseases), and outcomes (risk factors for COVID-19 and prognostic outcomes) (PECO) framework. To suit the context of our study, we replaced I (Intervention) in the usual PICO with E (exposures). In addition to the PECO inclusion criteria, only peer-reviewed published articles irrespective of language were included. Study designs of articles selected for further review and synthesis included case reports, case series, case-control, cross-sectional, cohort studies, and randomized control trials. Primary articles deemed eligible were also identified from the results section of systematic reviews in the database and selected for further review in our study. Articles were excluded if they involved other types of allergies (food allergy, drug allergy, skin allergy) and articles (reviews, book chapters, editorials, letters, and conference abstracts).

### Quality assessment/critical appraisal

Two reviewers independently evaluated the quality of the eligible articles using the appropriate quality assessment tools for each study design of these articles. The NIH Quality Assessment Tool for observational studies [16] was used for the study that was case series, case-control, cross-sectional, and cohort. Furthermore, the Joanna Briggs Institute (JBI) checklist [17] was used to appraise the case reports. Other two reviewers (KEO and NBHD) reviewed the critically appraised articles for any discrepancies. There were no disagreements between the reviewers during the assessment process.

### Data extraction

**Fig. 1:**
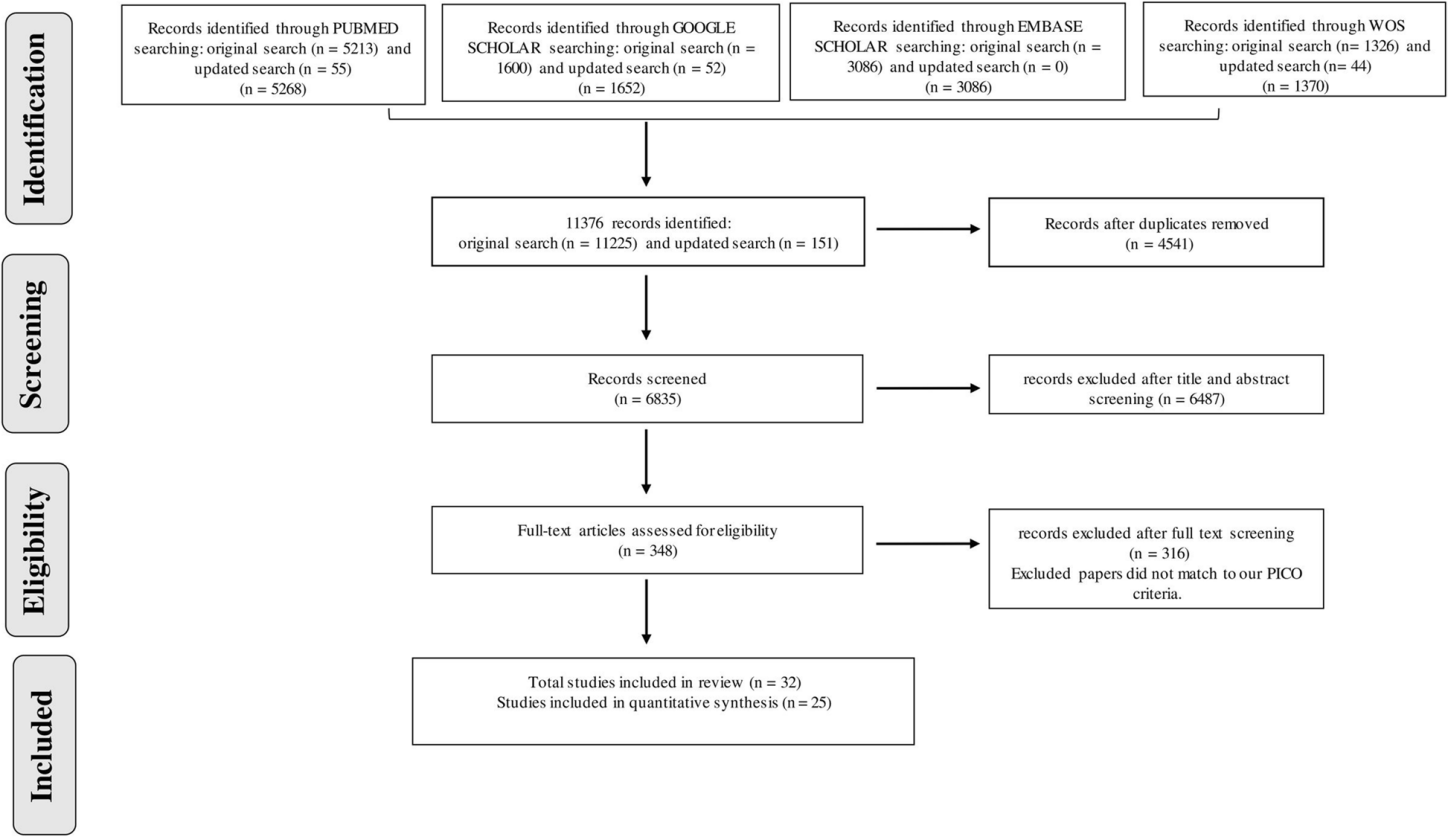
PRISMA flow diagram illustrating the search process which eligible articles were identified for data synthesis

The data extraction process was done according to the list of items detailed in the design in a data extraction rubric. The data extracted include author, year of publication, geographical setting, study objectives, study design, study population such as respiratory allergic patients, sample size, treatment for COVID-19 and respiratory allergy, and treatment outcomes/useful statistical findings in the study. There were no scenarios of missing information or difficulty with the retrieval of full text for eligible articles. The PRISMA flow diagram (Fig. 1) provides summaries of the methodological steps performed in this review.

### Data synthesis

The data synthesis first involved a summary of findings synthesized from the data extracted from eligible articles and presented in a tabular form ( Tables 1 and 2). To further quantitatively determine the prognosis of COVID-19 in patients with respiratory diseases, we carried out a meta-analysis to report point estimates and the confidence interval. The meta-analysis was performed using a random effects model because of heterogeneity in the eligible studies that were synthesized. Heterogeneity of individual studies was evaluated using the *I*^*2*^ statistics and was graphically presented using a forest plot.

**Table 1:** Summary description of study population in the synthesized articles

**S/N**	**Study authors**	**Study site and country**	**Study aim/** **objective**	**Study design**	**Race /Ethnicity**	**Participants of the study (sample size, age (years)—Male Gender No-- BMI, kg/m2)**	**Number and type of allergic respiratory patients** **(Reported allergen)**	**Phenotype of asthma** **(Asthma Control test - **FEV1%/FVC%)	**Other Comorbidities**	**Vaccination history (influenza, pneumococcal vaccines)**	**Smoking status**	**Implication of findings**
1.	Aghdam et al 2020 [18]	Ayatollah Moussavi Hospital, Zanjan, Iran.	To report a case of a child with asthma with an initialdiagnosis of COVID-19 pneumonia whose clinical courserevealed an underlying condition.	Case report	Iranian	7 years old boy.	1 (Allergic asthma)	Not reported	None	Not reported	Not applicable	In patients with COVID-19 and allergic asthma, other underlying hidden causes must be investigatedThis is because foreign body aspirationcontributed to the initial poor response to the treatment of this patient.
2.	Barsoum 2020 [19]	South West Acute Hospital, Ireland	To provide evidence to suggest that young people with asthma are at increased risk for COVID-19 infection	Case report	Not reported	12-year-old girl	Not reported	Not applicable	Asthma	Not reported	Not applicable	Oral corticosteroids used to management of asthma prolonged the duration of COVID-19 clearance. Thus, the investigation infers that asthma is a risk factor that can worsen prognosis in individuals infected with COVID-19 irrespective of age and should be further investigated through research.
3.	Renner et al. 2020 [20]	Helsinki University Hospital, Helsinki, Finland.	To report data on COVID-19 patients with severe asthma who are treated with monoclonal antibodies.	Case report	Finnish	41 years old male	1 (100%) asthmatic patients	Not reported	Not reported	Not reported	Not reported	Asthma is not associated with the severity of COVID-19.
4.	Schleicher et al. 2020 [21]	Wits Donald Gordon Medical Centre, Johannesburg, South Africa.	To describe the COVID-19 with other respiratory diseases.	Case report	African	53 years old man	1 (100%) asthmatic patients	Not reported	Pneumonia and Cytokine Release Syndrome.	Not reported	Not reported	This case study of severe Covid-19 with asthma, pneumonia, and Cytokine Release Syndrome showed some of the diagnostic and therapeutic challenges and controversies regarding the management of this novel and complex infection.
5.	Turbin et al. 2020 [22]	Rutgers New Jersey Medical School, Newark, New Jersey, USA.	To report a COVID-19 patient with other comorbidities	Case report	American.	12 and 15 years.	1 (50%) asthmatic patients	Not reported	Orbital cellulitis, sinusitis, and intracranialabnormalities	Not reported	Not reported	The COVID-19 may associate with other bacterial diseases.
6.	Vasconez et al. 2020 [23]	Miller School of Medicine, Miami, United States	To report COVID-19 with other comorbidities	Case report	American.	A 16 years old female.	1 (100%) asthmatic patients	Not reported	Severe diabetic ketoacidosis	Not reported	Not reported	Clinical suspicion of COVID should be heightened in children who present with unexplainedly severe diabetic ketoacidosis.
7.	Barroso et al 2020 [24]	Madrid, Spain	To report the prevalence of asthma and T2 diseases on a sample of hospitalized patients with COVID-19.	Case series	Not reported	Mean, or median age is not specified for all cases. But it was reported that the majority of the cases were females. No estimates were provided.	Of the 189 cases, 14 (7.4%) AR6 (3.2%) allergic asthma	Not reported	Most reported was diabetes, obesity, hypertension, hyperlipemia	Not reported	Quite a few were active smokers	There was no evidence in this case series that having allergic respiratory conditions with COVID-19 could predispose one to a risk of hospitalization or death.
8.	Bhatraju et al. 2020 [25]	University of Washington–Harborview Medical Center, USA	To describe the demographic characteristics, coexisting conditions, imaging findings, and outcomes among critically ill patients with Covid-19 in the Seattle metropolitan area.	Case series	Not reported	Mean (±SD) ageof the 24 critically ill cases were 64±18 years (range, 23 to 97) and63% were men.	Not reported	Not reported	Out of the 24 cases, 14(58%) DM, 5 (21%) CRD, and 3 (14%) hadasthma, Of total cases, 8 (33%) had morethan one coexisting condition.	Not reported	A total of 24 cases, 5 (22%) were current or former smokers	There was no further specific information like clinical management and treatment outcomes for the three asthmatic cases identified.
9.	Garg et al. 2020 [26]	Department of Health and Human Services regions, USA	Report an endemiological changes in COVID-19 new cases in the USA.	Case series (Retrospective)	Non-Hispanic white (white) (45.0%)non-Hispanic black (black) (33.1%)Hispanic (8.1%)Asian (5.5%)American Indian/Alaskan Native (0.3%)other or unknown race (7.9%)	Age (18-65 years) Males 49% Females 51%	27 (17.0%) asthmatic patients	Allergic	HTN (49.7%) Obesity (48.3%) Chronic metabolic disease (36.1%) DMus (28.3%) Chronic lung disease (34.6%)	NR	NR	Among patients aged 18–49 years, obesity was the most prevalent underlying condition, followed by chronic lung disease (primarily asthma) and diabetes mellitus.
10.	Guan et al 2020 [27]	Wuhan JinYinTan Hospital, China	To evaluate the risk of serious adverse outcomes in patients with COVID-19 by stratifying the comorbidity status.	Case series (Retrospective)	NR	Mean age 48.9 years Males 57.3% Females 42.7%	0	-	HTN (16.9%), CHD (3.7%) cerebrovascular diseases (1.9%) DM (8.2%) hepatitis B infections (1.8%) COPD (1.5%) CRD (1.3%) malignancy (1.1%) immunodeficiency (0.2%)	-	7%	Patients with comorbidities show poor outcomes than patients without comorbidities.
11.	Gold et al., 2020 [28]	Emory Decatur Hospital, Decatur, Georgia, USA	Report an endemiological change in COVID-19 new cases in Georgia, USA.	Case series	Black 81%Others 19%	Age (18-65 years) Males 49.5% Females 50.5%	32 (10.5%) asthmatic patients	NR	DM (39.7%) CHD (25.6%) CAD (11.5%) Congestive heart failure (10.8%) Arrhythmia (5.9%) Chronic lung disease (20.3%) COPD (5.2%) Severe obesity (12.7%) Immunocompromising conditions or therapies (9.2%) End-stage renal disease, on dialysis (5.2%) Liver disease (2.3)	NR	5.2%	Asthma associated with SARS-COV-2 may have a role in the severity of the disease.
12.	Otto et al., 2020 [29]	The Children’s Hospital of Philadelphia (CHOP) Care Network, Burlington, North Carolina, and Secaucus, New Jersey, USA	Understanding the prevalence and clinical presentation of COVID-19 in pediatric patients can help healthcare providers and systems prepare and respond to this emerging pandemic.	Case series (Retrospective)	White (49.8%) Black or African-American (29.6%) Asian or Asian Indian (3.2%) Multi-racial (3.6%) Other Races (13.8%) Not Hispanic or Latino (87.8%) Hispanic or Latino (10.2%) Not specified (2.0%)	Mean age 5.9 years Males 54.3% Females 45.7%	87 (20.5%) asthmatic patients	NR	NR	NR	NR	Most of the cases were mild, few children had the critical illness, and two patients died
13.	Takemoto et al., 2020 [30]	Departments of Health epidemiological reports, Brazil	To report the mortality data from Brazilian and compare to worldwide.	Case series	NR	Age (20-43 years) Females 100%	5 (25.0%) asthmatic patients	NR	Obesity Pyelonephritis CHD	NR	NR	There is one of the largest available series of maternal deaths due to COVID-19.
14.	Richardson et al., 2020 [31]	New York City Area the USA	To describe the clinical characteristics and outcomes of patients with COVID-19Hospitalized in a US health care system.	Case series	African American 1230 (22.6) Asian 473 (8.7) White 2164 (39.8) Other/multiracial 1574 (28.9)	5700 Female 2263 (39.7) Male 3437 (60.3). The median age of the total population 63 years	Asthma 479 (9%) BUT phenotype of asthma not reported	NR	Cancer 320 (6%) HTN 3026 (56.6%) CAD 595 (11.1) CHD 371 (6.9%) COPD 287 (5.4%) Obstructive sleep apnea 154 (2.9%) HIV 43 (0.8%) History of solid organ transplant 55 (1%) CRD 268 (5) End-staged 186 (3.5%) Liver disease Cirrhosis 19 (0.4%) Chronic HBV 8 (0.1%) HCV 3 (0.1%) Obesity (BMI ≥30) 1737 (41.7%) Morbid obesity (BMI ≥35) 791 (19.0%) DM 1808 (33.8%)	NR	Never smoker 3009 (84.4%)	This study provides characteristics and early outcomes of patients hospitalized with COVID-19 in New York.
15.	Argenziano et al. 2020 [32]	New York-Presbyterian/Columbia University Irving Medical Center, a quaternary care academic medical center in New York City, USA	To characterize patients with coronavirus disease 2019 (covid-19) in a large New York City medical center and describe their clinical course across the emergency department, hospital wards, and intensive care units	Cohort study	Asian, White, Hispanics/Latino and Blacks or African American	Median age 63.0 years (IQR: 50.0-75.0). Of a total of 1000 study population, males are 596. Median BMI for all patients was 28.6 (IQR: 25.2-33.1)	Not specified However, what was reported was that there were 113 asthmatic patients. It was not reported if this asthma was an allergic asthma	Not reported	Mainly cardiovascular with HTN as most prevalent, followed by diabetes. In addition, some had cancer, cirrhosis, viral hepatitis, renal disease, and HIV	Not reported	About 181 are active smokers but not specified, which among these were the asthmatic.	Of the total 1000 study population, it would have been interesting to specifically profile and report those who had allergic respiratory conditions, their treatment, and outcomes
16.	Docherty et al., 2020 [33]	England, Wales, andScotlandUK	To depict the clinical features of COVID-19 patients during the first wave in the United Kingdom.	A prospective observational cohort study	NR	The total study population was 20133, of which Male 60%, *n *=120;68 Female 40%, *n *= 8065. The median age of the total population was 73 years	Asthmatic patients were 2540 (14.5); Male 1192 (11.4) And Female 1348 (19.1). However, phenotype of asthma not reported	NR	Malignancy1743 (10.0%) CHD 5469 (30.9%) DM without complications 3650 (20.7%) DM with complications 1299 (7.4%) non-asthmatic CPD 3128 (17.7%) CRD 2830 (16.2%) Obesity1685 (10.5%) HIV 83 (0.5%) Moderate or severe liver disease 310 (1.8%) Mild liver disease 281 (1.6%) Chronic hematological disease 693 (4.0%) Rheumatological disorder 1696 (9.8%) Malnutrition 396 (2.4%)	NR	Never smoked 8968 (63.2) Former smoker 4364 (30.8) Yes 852 (6.0)	This study showed the importance of pandemic preparedness to reduce the mortality rate.
17.	Du et al., 2020 [34]	Wuhan China	To investigate the clinical characteristics ofCOVID-19 children with different severities and allergic status.	Retrospective Cohort Study	NR	Total population182. Male 120 (65.9%), Female 62 (34.1%). The median age of the total population was 6 years.	Asthma + urticaria +drug allergy 1 (2.3%) AR 28 (65.1%) AR + drug allergy 5 (11.6%) AR + AD 1 (2.3%) AR + food allergy 1 (2.3%) AR + food allergy + drug allergy 1 (2.3%) Asthma + urticaria +drug allergy 1 (2.3%) AD 3 (7.0%) Penicillin 10 (23.3%) Mango 1 (2.3%) Egg 1 (2.3%) Dust mite 1 (2.3%)	Allergic	Medical history except allergic. Nonallergic patients 8 (18.6%) Nonallergic patients 24 (17.3%)	NR	NR	The clinical course in Pediatric has a mild clinical course; also, there was no difference in disease between allergic and nonallergic COVID-19 children.
18.	Grandbastien et al. 2020 [35]	Chest Diseases Department of Strasbourg University Hospital, France	To assess the frequency of asthma exacerbation in patients with asthma hospitalized for SARS-CoV-2 pneumonia and compare symptoms and laboratory and radiological findings in patients with and without asthma with SARS-CoV-2 pneumonia.	Cohort study	NR	Mean age 63.5 years. Males 62.3% Females 37.7%	23 (21%) asthmatic patients	Allergic	Obesity (39.6%) HTN (42.5%) DM (21.7%) CHD (5.7%) CRD (4.8%) Lung cancer (6.6%) Obstructive sleep apnea (13.2%)	NR	33 (31.1)	The study result found that the patient with asthma has no risk for SARS-COV-2 severe symptoms; also, SARS-CoV-2 pneumonia did not induce severe asthma symptoms.
19.	Ibrahim et al. 2020 [36]	The Royal Children’sHospital, Melbourne, Australia.	To presentearly data on Australian children.	Retrospective cohort study	NR	Mean age 13.1yearsMales 25%Females 75%	1 (25%) asthmatic patient.	NR	NR	NR	NR	The prevalence of SARS-COV-2 was low in the children; also, asthma had a low prevalence.
20.	Jacobs et al., 2020 [37]	USA	To review early clinical experience with the use of extracorporeal membrane oxygenation in patients with confirmed COVID-19 and pulmonary disease.	Cohort study	NR	Total population 32. Male 22 (68.8%), Female 10 (31.2%). The median age of the total population was 52 years	There was only 3 (9.4%) asthmatic patient but phenotype **NOT** reported	NR	Cancer 3 (9.4%) DM11 (34.4%) CHD4 (12.5%) Obesity Yes 14 (43.8)	NR	NR	Extracorporeal membrane oxygenation needs more studies to confirm using in COVID-19.
21.	Kim et al., 2020 [38]	Korea	To investigate the clinical course and outcomes of COVID-19 from early cases in Korea.	Retrospective multicenter Cohort Study	NR	Total population 28. Male 15 (53.6%), Female 13 (46.4%). Median age of total population was 40 years	There was only 1 (3.6%) asthmatic patient but phenotype **NOT** reported	NR	Cancer 1 (3.6%) HTN 0 Dyslipidaemia 0 DM without complication 2 (7.1%) CHD 0 CRD 0COPD 0 Liver disease, mild 1 (3.6%) HIV 0	NR	5 (18.5%)	The asthmatic patient showed bilateral CT only, was isolated without oxygen requirement, and received lopinavir/ritonavir antiviral therapy
22.	Li et al., 2020 [39]	China	To evaluate the severity on admission, complications, treatment, and outcomes of patients with COVID-19.	Retrospective cohort study	NR	The total study population was 548 Mean age 60 years, 279 of 548 (50.9%) were males, mean BMI was 24.7 (kg/m2)	5 of 548 (0.9%) patients had asthma Phenotype **NOT** reported	NR	Tuberculosis 9 of 548 (1.6%), diabetes 83 of 548 (15.1%), HTN 166 of 548 (30.3%), CAD 34 of 548 (6.2%), hepatitis B 5 of 548 (0.9%), CRD 10 of 547 (1.8%), and tumor 24 of 513 (4.7%)	NR	Never smokers 452 of 544 (83.1%), Former smokers 51 of 544 (9.4%), Current smokers 41 of 544 (7.5%)	Older patients with hypertension and high lactate dehydrogenase need early support to reduce the severity of the disease.
23.	Mahdavinia et al., 2020 [40]	Rush University Medical Center. USA	To report the result of the role of asthma in the outcome of COVID-19 in a large cohort of COVID-19 positive patients.	Cohort	African American 59.7% Asian 8.7% White non-Latino 11.3% White Latino 5.1%	Age (18-65 years) Males 66.8% Females 33.2%	241 (25.7%) asthmatic patients	NR	Obesity	NR	NR	Preexisting asthma is a risk for COVID-19 and predictor of intubation duration in COVID-19, especially in patients less than 65 years.
24.	Singer et al., 2020 [41]	New York Hospital and Predictors of ICU Care, USA	To report the COVID-19 cases and outcomes.	Retrospectivecohort	White (42%) Black (7%) Asian (3%) Other (1%) Unknown (47%) Hispanic (37%)	Mean age 50 years Males 54% Females 46%	106 (6%) asthmatic patients	NR	HTN (28%) DM (15%) CAD (7%) COPD (4%) CHF (3%) Cancer (4%) Immunosuppressed (4%) CKD (5%)	-	6%	9% of COVID-19 patient need immediate ICU, and 13% need mechanical ventilation within 2 to 3 days.
25.	Borba et al., 2020 [42]	Care facility in Manaus, Brazilian Amazon, Brazil	To evaluate the safety and efficacy of 2 CQ dosages in patients with severe COVID-19.	RCT	White (21%) Mixed (71.6%) Black (7.4%) Pregnant (10%)	Mean age 51.1yearsMales 75.3%Females 24.7%	4 (7.4%) asthmatic patients	Not reported	HTN (45.5) DM (25.5) Alcohol use disorder (27.5) HD (9.1) CRD (7.4) Rheumatic diseases (5.5) Liver diseases (3.6) Tuberculosis (3.6)	Not reported	4 (8.3%)	This study suggested that the higher chloroquine diphosphate is not recommended in COVID-19 patients.
26.	Chao et al., 2020 [43]	Tertiary Care Medical Center in New York City.	To describe the clinical profiles and risk factors for COVID-19 patients.	Cohort study	White 1 (3) Black 3 (9.1) Latino 26 (78.8) Other 3 (9.1)	Mean age of 9.4 years Males 69.6% Females 30.4%	11 (23.65%) asthmatic patients	Not reported	Obesity (27.3%) Immunosuppressed 1 (3) Seizure disorder 1 (3)	Not reported	Not reported	pediatric with complications as diabetes need early admission in the intensive care unit.
27.	Chhiba et al., 2020 [44]	University Feinberg School of Medicine, Chicago.	To determine theprevalence of asthma among patients with COVID-19	Retrospective cohort	Non-Hispanic African American 358 (23.5) Non-Hispanic white 643 (42.1) Hispanic or Latino 324 (21.2) Non-Hispanic Asian 70 (4.6) Other 201 (13.2)	Mean age 55 years Males 47% Females 53%	220 (14.4%) asthmatic patients	Not reported	Not reported	Not reported	53 (3.5)	This study found that asthma prevalence was 14% in a cohort ofpatients with COVID-19.
28.	Desir et al., 2020 [45]	The New York-Presbyterian hospital network.	The study objective was to determine whether underlying asthma was associated with poor outcomes among COVID-19 patients.	Retrospective cohort	Black 238 (21) White 218 (19) Asian 14 (1) Other 384 (34)	Mean age 51.5 years Males 98% Females 2%	163 (12.55%) asthmatic patients	Not reported	Obese 445 (39) Other 593 (52)	Not reported	55 (3%)	Asthma diagnosis was not associatedwith worse outcomes in COVID-19 patients.
29.	Salacup et al., 2020 [46]	Einstein Healthcare Network is an inner-city urban community hospital in Northern Philadelphia	To describe the demographics and clinical factors of COVID‐19 patients of a minority population in an underserved area.	Retrospective cohort	Not reported	Mean age 66 years Males 49% Females 51%	18 (7%) asthmatic patients	Not reported	COPD 30 (12) CHD 35 (15) Atrial fibrillation 24 (10) Liver cirrhosis 8 (3) DM 118 (49) CRD 42 (17) CAD 45 (19) HTN 180 (74) Obesity 97 (40)	Not reported	Not reported	The mortality rate significantly increased with old age.
30.	Schultze et al., 2020 [47]	London, UK.	To assess the association between inhaled corticosteroids and COVID-19-related death among people with COPD or asthma.	Cohort study	White (75%) Mixed (0.2%) Asian or Asian (0.6%) Black (0.2%) Other (0.3%) Unknown (23.7%)	Mean age 31 years Males 55% Females 45%	Not reported	Not reported	Not reported	Not reported	17 268 (39·9%)	This study results do not support the role of inhaled corticosteroids in protecting asthmatic patients against COVID-19.
31.	Yang et al., 2020 [13]	South Korea	To determine the association of allergicdisorders with the likelihood of COVID-19.	A nationwide cohort study	Not reported	Mean age 49 years Males 47.4% Females 52.6%	725 (9.8%) asthmatic patients. 4210 (57.3) allergic rhinitis.	Not reported	Not reported	Not reported	Not reported	Asthma and allergic rhinitis confer risk for COVID-19.
32.	Zhang et al., 2020 [48]	Hospital of Wuhan, China.	To investigate the clinical characteristic and allergy status of COVID-19 patients	Cohort study	Not reported	Mean age 57yearsMales 50.7%Females 49.3%	0	Not reported	HTN 42 (30.0) DM 17 (12.1) Arrhythmia 5 (3.6) Urolithiasis 3 (2.1) Stroke 3 (2.1)CRD 2 (1.4) Aorta sclerosis 2 (1.4) COPD 2 (1.4)	Not reported	2 (1.4%)	Allergic diseases, asthma, and COPD are not risk factors for COVID-19.

Allergic rhinitis, *AR* Atopic dermatitis, AD; *HT* hypertension, *DM* diabetes mellitus, *CAD* coronary artery disease, *CHD* chronic heart disease, *COPD* chronic obstructive pulmonary disease, *CRD* chronic renal disease, *NR* not reported

**Table 2:** Summary of findings and implications in synthesized articles

S/N	Study authors	Corticosteroids, bronchodilators, leukotriene antagonist administration, type, and duration	Prognostic outcome	Implication of findings
1.	Aghdam et al 2020 [18]	In this case report of a 7-year-old boy who presented with 2 years history of allergic asthma, exposure to inhaled corticosteroids based on this history was reported but duration not specified. During his 8 day hospitalization for COVID-19, there was administration of fluticasone sprays along with intravenous hydrocortisone for at least 6 days.	Discharged and well. Initially, patient responded poorly to treatment until foreign body aspiration was identified.	Other underlying hidden causes other than COVID-19 must not be neglected in patients with concurrent COVID-19 and allergic respiratory conditions like allergic asthma
2.	Barroso et al 2020 [24]	In this case series, LABA, SABA and ICS were administered to 11 asthmatic patients out of whom 6 had allergic asthma. For all 11 asthmatic patients, six had intermittent-asthma using short-acting-ß2-agonist and five with moderate-asthma on treatment with long-acting-ß2-agonist combined with inhaled glucocorticoid (LABA/GCI). Two of them with low-dose-LABA/GCI (one had prednisone 5mg/daily for rheumatoid arthritis) and the other three with medium-dose LABA/GCI (one had Antileukotrienes montelukast 10 mg/daily) Only one from the five patients with moderate-asthma had good compliance with treatment. Ten from the eleven had well controlled asthma, and one had partially controlled asthma (medium-dose-LABA/GCI and montelukast).	Two (2) patients had an asthma exacerbation on admission for COVID-19. One of them died in ICU due to complication of orotracheal-intubation, a woman of 70 years with allergic moderate-asthma on treatment with medium-dose-LABA/GCI and montelukast, with bad compliance of inhaled treatment and other comorbidities (severe sleep-apnea-hypopnea-syndrome, obesity); she was treated with LABA-GCI and systemic GC during hospitalization. The second patient with asthma exacerbation was a woman of 42 years with allergic moderate-asthma and obesity, active smoker, type 2 diabetes, and bad compliance with inhalation therapy; she received inhaled LABA-GCI during hospitalization but not systemic GC.	The authors are of the opinion that the prognostic outcome earlier described can be attributed to underlying comorbid conditions these cases had. They recommended for these findings to be confirmed by cohort studies with larger sample size of respiratory allergic patients with COVID-19.
3.	Barsoum 2020 [19]	In this case report of a 12-year-old girl with history of asthma but phenotype not reported, oral corticosteroids was administered the first day she came to the accident and emergency department and later discontinued the next day once the diagnosis of COVID-19 was confirmed.	Case improved and was discharged after 2 days.	Underlying comorbidity such as asthma may increase risk of susceptibility to COVID-19. This is because the oral corticosteroids used to management of asthma prolonged the duration of COVID-19 clearance. The authors therefore recommend that clinical presentations of COVID-19 in children be critically reviewed to improve treatment outcomes.
4.	Bhatraju et al., 2020 [25]	In this case series of 24 critically ill cases, the 3 asthmatic cases received as an outpatient, systemic glucocorticoids for a presumed asthma exacerbation before becoming critically ill.	These 3 patients then presented to the hospital again, with severe respiratory failure requiring invasive mechanical ventilation.	The implications of this are uncertain and they recommend further research is necessary to determine the role of systemic glucocorticoids in patients with COVID-19 infection.
5.	Grandbastien et al., 2020 [35]	12 patients were not received any inhaled corticosteroid, and 11 patients were received inhaled corticosteroids combined with bronchodialators (only 1 patient was treated with biotherapy and oral corticosteroids).	Among patients with asthma, 14 patients were well controlled, 6 patients were partially controlled, and 2 patients were noncontrolled.	This suggests that the risk factors for hospitalization in their patients were related more to the risk factors of SARS-CoV-2 pneumonia (e.g., hypertension, obesity, diabetes, tobacco smoke, and obstructive sleep apnea) than to asthma. SARS-CoV-2 pneumonia did not induce the severe asthma symptoms. pollen allergy appeared not to be the reason for asthma exacerbation in our patients.
6.	Desir et al., 2020 [45]	Systemic corticosteroids 44 (27%)	Hospitalized COVID-19 patients with asthma were more frequently treated with systemic steroids compared with those without asthma (27% vs 17%; *P* < .01).	The implications of this are uncertain at this time and may have favorably or adversely affected outcomes in these patients.
7.	Chhiba et al., 2020 [44]	Outpatient *N*=105 No ICS 57.1% ICS 14.3% ICS/LABA 28.6% Inpatient - no ICU *N*=96 No ICS 50% ICS 9.4% ICS/LABA 40.6% Inpatient - ICU *N*=19 No ICS 31.6% ICS 10.5% ICS/LABA 57.9% Only 15 patients were prescribed systemic corticosteroids before diagnosis Outpatient (*N*= 7), inpatient - no ICU (*N*= 8) Inpatient - ICU (*N*= 0). Only 1 patient was receiving an asthma-related biologic (omalizumab). This patient required an ICU stay and was intubated for COVID-19 but was successfully discharged after 16 days of hospitalization.	Systemic corticosteroid use before COVID-19 diagnosis was not different between the outpatient and inpatient managed subgroups The use of ICS did not increase or decrease the risk of COVID-19 hospitalization in patients with asthma and COVID-19 (RR, 1.47; 95% CI, 0.93–2.32).	COVID-19-associated level of care (ICU vs non-ICU) was not significantly different between patients prescribed ICS or ICS/LABA and those not on ICS or ICS/LABA. Similarly, the use of inhaled corticosteroids with or without systemic corticosteroids was not associated with COVID-19-related hospitalization.

*AR* allergic rhinitis, *AD* atopic dermatitis, *HT* hypertension, *DM* diabetes mellitus, *CAD* coronary artery disease, *CHD* chronic heart disease, *COPD* chronic obstructive pulmonary disease, *CRD* chronic renal disease, *NR* not reported

## Results

The literature search retrieved 11,376 articles and 4541 duplicate studies were removed. After screening of titles and abstracts of the total 6835 articles after duplicates have been removed, we excluded 6487 studies that were not relevant to our inclusion criteria. We further conducted a full-text screening of the remaining 348 articles and, this resulted in the identification of 32 studies as eligible for inclusion in our systematic review (Fig. 1).

### Characteristics and quality of included studies

The summary and baseline characteristics of synthesized studies and the therapeutic management for respiratory allergic patients infected with COVID-19 are shown in Tables 1 and 2, respectively. According to the NIH Quality Assessment tool [16], the quality assessment of the included studies ranged between good and fair quality.

### The prevalence of respiratory allergy in COVID-19 patients

**Fig. 2:**
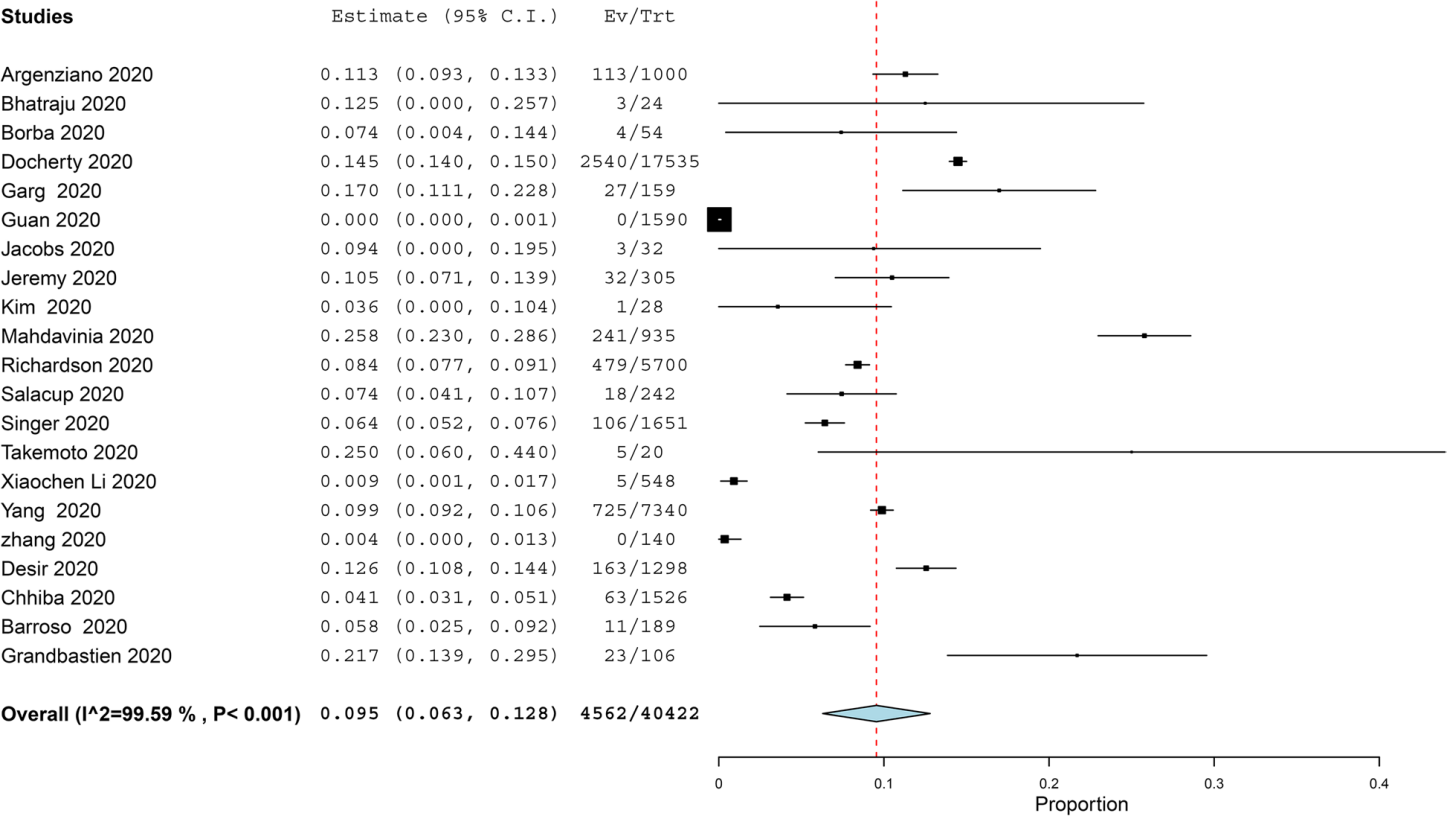
The prevalence of asthma in old COVID-19 patients

**Fig. 3:**
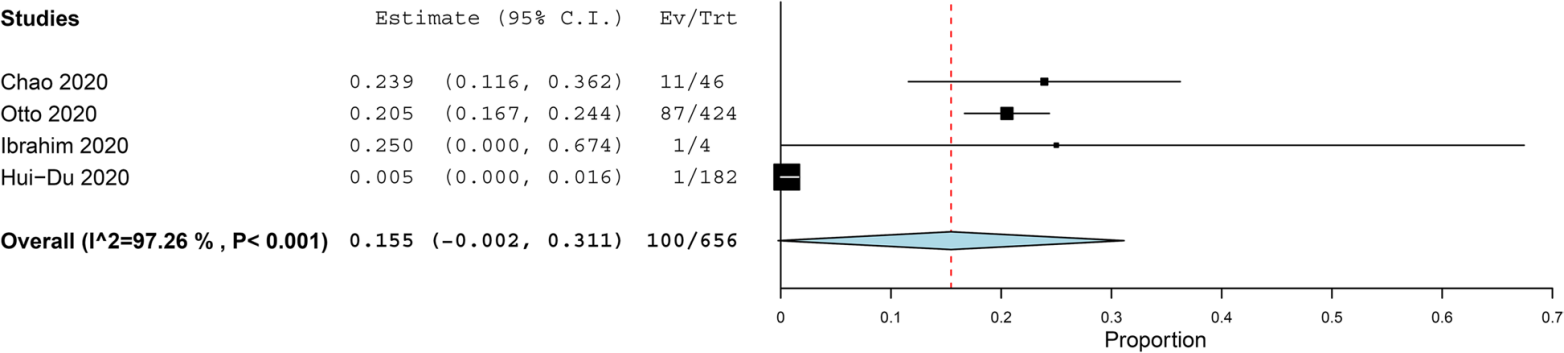
The prevalence of asthma in young COVID-19 patients

**Fig. 4:**
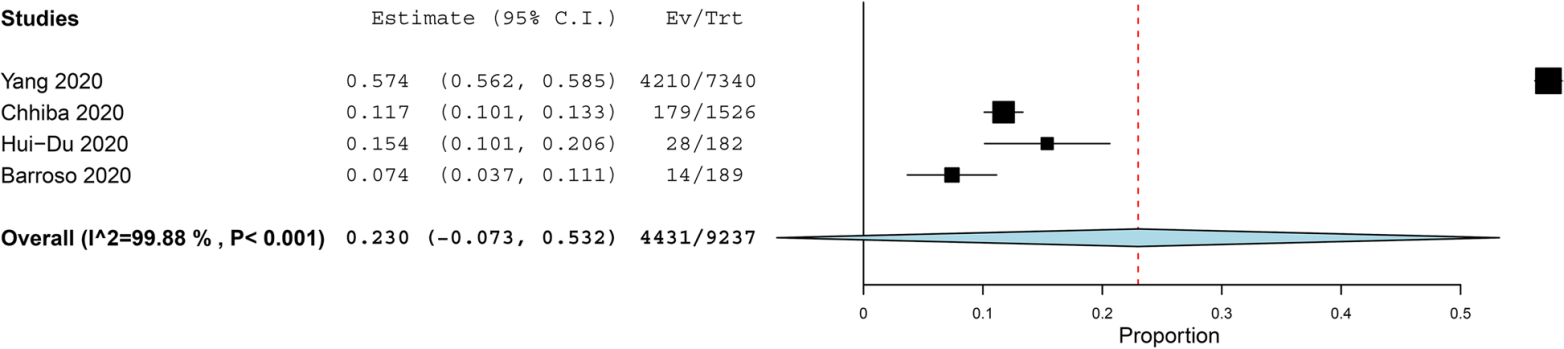
The prevalence of allergic rhinitis in COVID-19 patients

The prevalence of asthma in COVID-19 patients more than 11 years old was reported in 21 studies with 40,422 COVID-19 patients, under random effect model, the overall prevalence of asthma was 9.5% of COVID-19 patients with 95%, CI = 0.063, 0.128, *P* < 0.001. While the prevalence of asthma in COVID-19 patients under 11 years old was reported in 4 studies with 656 COVID-19 patients, under random effect model, the overall prevalence of asthma was 15.5% of COVID-19 patients with 95%, CI = − 0.002, 0.311, *P* < 0.054. The pooled results for the prevalence of asthma in COVID-19 patients above and under 11 years old are presented in Figs. 2 and 3, respectively. In addition to this, the pooled effect estimate demonstrated that there is no significant difference in association between COVID-19 with allergic asthma and non-allergic asthma (RR = 0.61, 95% CI 0.35, 1.06, *p* = 0.08), and this pooled result was homogenous (*P* = 0.05, *I*^2^ = 47%) as illustrated in Fig. 4. The prevalence of allergic rhinitis between COVID-19 patients was reported in 4 studies with 9237 COVID-19 patients, under random effect model; the overall prevalence of allergic rhinitis was 23% of COVID-19 patients (95%, CI = − 0.073, 0.532, *P* < 0.136) as displayed in Fig. 4.

### The prognostic outcomes for COVID-19 infection in asthmatic patients

**Fig. 5:**
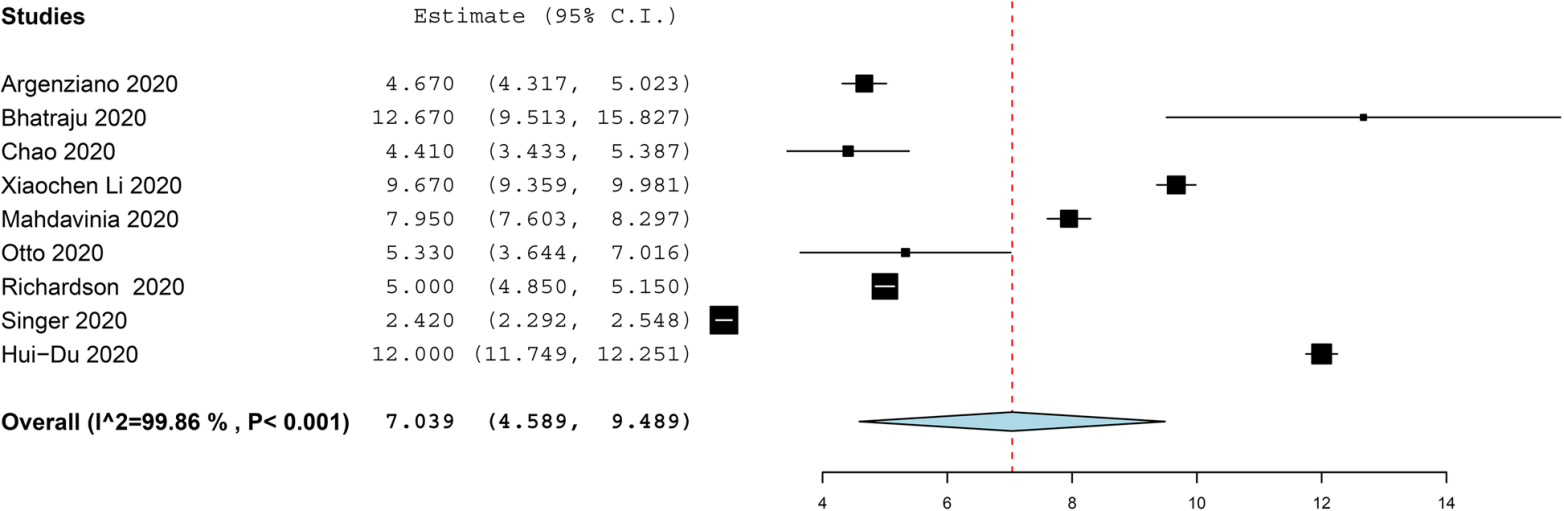
The hospitalization time in COVID-19 patients

**Fig. 6:**
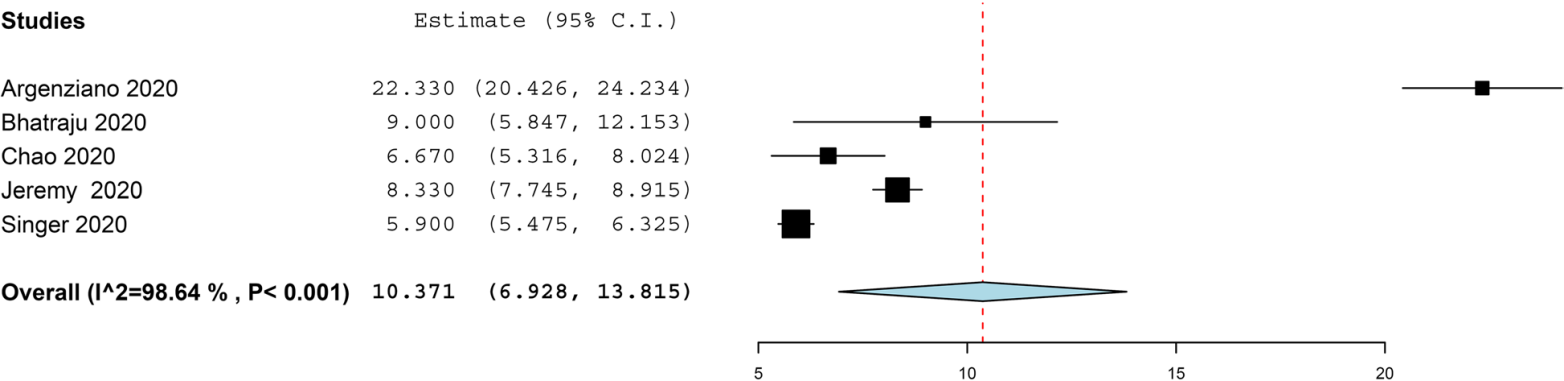
The intensive care unit time in COVID-19 patients

**Fig. 7:**
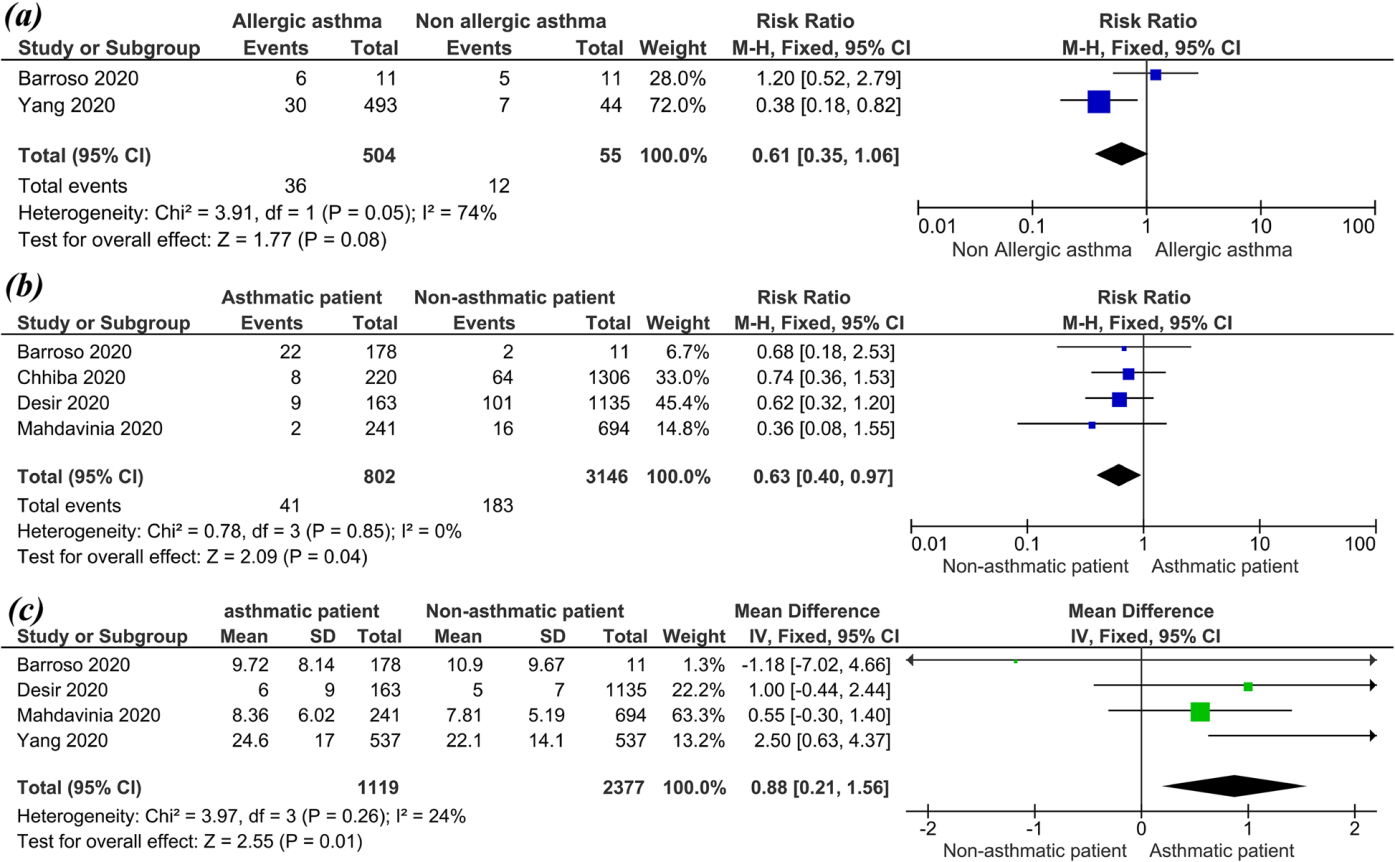
**a** The prevalence of COVID-19 in allergic and non-allergic asthmatics patients. **b** The mortality rate in asthmatics COVID-19 patients compared with non-asthmatics COVID-19 patients. **c** The hospitalization time in asthmatics COVID-19 patients compared with non-asthmatics COVID-19 patients

Nine (9) studies with 7880 COVID-19 patients reported that the mean duration of hospitalization time in COVID-19 patients was (estimated mean = 7.039, 95%, CI = 4.589, 9.489, *P* < 0.001), under the random effect model. the pooled result was heterogeneous (*I*^2^ = 99.68%, *P* < 0.001) (Fig. 5). Five (5) studies with 869 COVID-19 patients reported that the mean duration of ICU staying time in COVID-19 patients was (estimated mean = 10.37, 95%, CI = 6.928, 13.815, *P* < 0.001), under the random effect model. the pooled result was heterogeneous (*I*^2^ = 98.64%, *P* < 0.001) (Fig. 6). The pooled effect estimate showed that mortality rate significantly reduced by 30% in COVID-19 patients with asthma than in patients without asthma (RR = 0.63, 95% CI 0.40, 0.97, *p* = 0.04). There is no significant difference in associated between COVID-19 with allergic asthma and non-allergic asthma (RR = 0.61, 95% CI 0.35, 1.06, *p* = 0.08). These pooled results were homogenous (*P* = 0.05, *I*^2^ = 47%) (Fig. 7a).

The pooled effect estimate showed that hospitalization time significantly increased with asthmatic patients more than non-asthmatic patient (mean difference = 0.88, 95% CI 0.21, 1.56, *p* = 0.01). The pooled results for mortality rate and hospitalization time for COVID-19 infection in asthmatic patients were homogenous as presented in Fig. 7b, c, respectively.

## Discussion

### Principal findings

The present systematic review collects evidence from 32 studies that provide information about the prognostic outcome of COVID-19 in respiratory allergic patients (asthma & allergic rhinitis. The pooled results display no significant difference between the prevalence of COVID-19 with allergic asthma and non-allergic asthma. Asthma is characterized by chronic inflammation, hyper responsiveness of respiratory airways, mucus overproduction, and remodeling [49]. Allergy has been involved in 50–80% of asthma and the roughly 50% of severe asthma [50], though non-allergic asthma has been implicated in 10–33% of asthmatic individuals. The mechanism of allergic asthma has largely been associated with TH2 inflammation that is exemplified by high levels of eosinophils, IgE, and cytokines, such as IL-4, IL-5, IL-13, and IL-9 [51]. Compared with this allergic mechanism, TH1 response which involves the stimulation of neutrophils and mast cells has been characterized nonallergic asthma [52]. Together respiratory infections, failure in resolution of inflammation, and stimulation of IL-17 pathway attribute to neutrophilic inflammation [53]. In the included Korean nationwide cohort, allergic asthma patients were not diagnosed by their medical history, including laboratory data (e.g., IgE levels) but they were defined by International Classification of Disease codes, which may have miscaptured data, and allergic asthma was demarcated as asthma with at least one further allergic disorder (atopy or allergic rhinitis), while asthma without any atopic disorder was defined as nonallergic asthma [13].

### Strengths and limitations

This review affords up-to-date results of the risk of respiratory allergic disease in patients with COVID-19. To the best of our knowledge, this is the first systematic review focused on the prevalence and outcome of COVID-19 infection in allergic asthma and allergic rhinitis patients. These studies were generated from several countries. However, this meta-analysis had some limitations. First, while only two studies described the phenotype of asthmatic patients, others offered no information. Also, there were no data on asthma control due to a lack of lung function tests which were not performed due to restrictions recommended during COVID-19. Second, data on allergic rhinitis was sourced only from three studies, other studies combined this data with other allergies (food allergy, eczema). Third, there is no detailed information on asthma severity, systemic antihistamines, leukotriene antagonists, and allergen immunotherapy displayed in the identified studies which keep us from further conclusions about their role in COVID-19 prognosis. Fourth, the pooled results of the prevalence of allergic rhinitis and asthma between COVID-19 patients were heterogenous. This might be related to methodological discrepancies in the sampling criteria and study design across the studies included in this part of the analysis.

### Comparison with other studies

Regarding the outcome of COVID-19 infection in asthmatics patients, the pooled effect estimate showed that mortality rate was significantly reduced by 30% in COVID-19 patients with asthma than in patients without asthma (RR = 0.63, 95% CI 0.40, 0.97, *p* = 0.04). Our study findings are similar to results from a previously published meta-analysis which reported the risk of mortality in patients with COVID-19 with asthma was (RR = 0.87, 95% CI 0.69, 1.09, *p* = 0.24) [54]. Their results were not significant as they included only three studies and used random effect model. On the other hand, no significant effect was reported by Wang et al. [55] (4 studies) for the mortality risk (OR = 0.96; 95% CI 0.70–1.30; *I*^2^ = 0%; *p* = 0.79). Among the four included studies in the current analysis, Desir et al. weighted 45.4%, noted that the prevalence of asthma as a comorbidity in severe COVID-19 seems to be parallel to that of coexistent conditions such as hypertension, diabetes, and hyperlipidemia.

There are various justifications for this result. Earlier, the overall prevalence of asthma in COVID-19 patients under 11 years old and more than 11 years old patients were 15.5% and 9.5% respectively. The second explanation is that asthmatic patients were adherent to home isolation precaution during the COVID-19 epidemic as they were known as a high-risk group. Moreover, the hospitalized asthmatic patients were presented early to the hospital and were received an aggressive and timely management. The low prevalence of asthma may be attributed to the low risk of asthmatic patients to COVID-19 infection. Jackson et al. observed that allergic asthmatic patients have reduced expression of ACE2 in respiratory epithelial cells [56]. This can be attributed to the TH2 inflammatory pathway and asthmatic medications (ICS alone or with bronchodilators) which inhibit viral replication [57, 58]. These observations may explain the low mortality risk in asthmatics patients with COVID-19 infection.

The hospitalization time significantly increased with asthmatic patients more than non-asthmatic patient (MD = 0.88, 95% CI 0.21, 1.56, *p* = 0.01). Our finding could be clarified by the fact that the symptoms of asthma are exacerbated by respiratory viral infections and the management of asthma becomes more complicated during the COVID-19 pandemic [49]. The respiratory viruses penetrate the epithelium of the respiratory airway and elicit local inflammation, which disrupts the bronchial defense system [59]. There are several cytokines induced by a viral infection that play role in the exacerbation of asthma. Secretion of IL-25 and IL-33 in epithelial cells stimulates TH2 pathways to cause increased mucin production, eosinophilia, and secretion of proinflammatory cytokines (i.e., IL-4, IL-5, and IL-13) [60]. Interferons (IFNs) engage in a pivotal role in antiviral and allergic responses. Earlier studies have revealed that IFN secretion by respiratory epithelial cells and plasmacytoid dendritic cells (pDCs) is reduced in asthma [61]. Additionally, IgE cross-linking reduces antiviral responses through inhibition of pDC maturation, IFN-α response, and TLR-7 upregulation [62].

Concerning hospitalization and mortality in allergic rhinitis patients with COVID-19 infection, we could not do analysis as there is not enough data. Only included cohort studies assumed that allergic rhinitis showed a trend toward lower hospitalization, although not statistically significant (RR, 0.83;95%CI,0.64-1.07) [44]. There were seven synthesized articles that mentioned corticosteroids, bronchodilators, leukotriene antagonist therapy, and their outcome in asthmatic COVID-19 patients. They included 2 case reports and 1 case series that noted that prognostic outcome of COVID-19 infection can be attributed to underlying comorbid conditions and hidden causes other than COVID-19 in patients with concurrent COVID-19 and respiratory allergy [18, 19, 24]. In a French cohort study, they concluded that that the risk factors for hospitalization in their asthmatic patients were related more to the risk factors of SARS-CoV-2 pneumonia (e.g., hypertension, obesity, sleep apnea diabetes, smoking) than to asthma [63]. Bhatraju et al. could not draw a conclusion regarding the role of systemic glucocorticoids in patients with Covid-19 infection but recommended further research [25]. However, Chhiba et al., 2020, concluded that the use of ICS with or without systemic corticosteroids was not associated with COVID-19-related hospitalization [44].

## Conclusions

Based on current findings, there was little evidence on therapeutic management of respiratory allergic patients infected with COVID-19 and the impact on prognostic outcomes. Consequently, it is critical that asthmatic patients should continue to administer medications prescribed to maintain asthma control regularly, in particular, ICS, long-acting bronchodilators, antileukotrienes drugs to avoid complications as increased hospitalization time. Further investigation is needed to determine the role of asthma medications and immunotherapy in the outcome of COVID-19 infection in asthmatic patients. In addition, the association of severe COVID-19 with other risk factors in asthmatic patients should be the topic of future studies.

## Data Availability

Not applicable

## Abbreviations

Th2: T helper 2
ACE2: Angiotensin-converting enzyme-2
TMPRSS2: Transmembrane protease, serine2
ICSs: Inhaled corticosteroids
PROSPERO: Prospective Register of Systematic Reviews
MeSH: Medical Subject Headings
